# Genome Assemblies for 11 *Yersinia pestis* Strains Isolated in the Caucasus Region

**DOI:** 10.1128/genomeA.01030-15

**Published:** 2015-09-17

**Authors:** Ekaterine Zhgenti, Shannon L. Johnson, Karen W. Davenport, Gvantsa Chanturia, Hajnalka E. Daligault, Patrick S. Chain, Mikeljon P. Nikolich

**Affiliations:** a National Center for Disease Control and Public Health (NCDC), Tbilisi, Georgia; b Los Alamos National Laboratory (LANL), Los Alamos, New Mexico, USA; c Walter Reed Army Institute of Research (WRAIR), Silver Spring, Maryland, USA

## Abstract

*Yersinia pestis*, the causative agent of plague, is endemic to the Caucasus region but few reference strain genome sequences from that region are available. Here, we present the improved draft or finished assembled genomes from 11 strains isolated in the nation of Georgia and surrounding countries.

## GENOME ANNOUNCEMENT

At the crossroads of Asia and Europe, along the Great Silk Road, Georgia and much of the Caucasus region have been exposed to many epidemics, including plague, which was first mentioned in the 11th century (1). Plague, a global zoonotic disease that has had devastating effects on human populations in three major pandemics, is caused by the Gram-negative coccobacillus *Yersinia pestis*. This disease has impacted the history of the Caucasus region, and unique variants that are known to circulate in the region have been characterized; however, few reference genomes are publicly available (2–4). The strains sequenced in this effort were selected from a historic collection of *Y. pestis* isolates from the Caucasus region preserved in the repository of the National Center for Disease Control and Public Health of Georgia.

High-quality genomic DNA was extracted from purified isolates of each strain and draft sequence data generated using a combination of Illumina and 454 technologies (5, 6). For each genome, we constructed and sequenced an Illumina library of 50 to 151 bp reads at high coverage (57- to 995-fold coverage), a 454 Titanium library of unpaired reads (17- to 42-fold coverage), and, excepting *Y. pestis* strains 1670 and 14735, a 454 Titanium long-insert paired-end library (insert size, 3.8 to 9.1 Kb with 2.1- to 21-fold genome coverage). PacBio long reads were generated for *Y. pestis* strain 790, with an average subread length of 3.5 Kb and 108-fold coverage (7). The 454 data sets were assembled together in Newbler (Roche), and the consensus sequences were computationally shredded into 2-Kbp overlapping fake reads (shreds). The raw Illumina reads were assembled in Velvet, and the Velvet consensus sequences were computationally shredded into 1.5-Kbp overlapping shreds (8). For *Y. pestis* strain 790, Illumina and 454 long-insert data were also assembled together with AllPaths, and the PacBio data were assembled with HGAP (9, 10); the consensus sequences for both assemblies were computationally shredded into 10-Kbp overlapping shreds. We then integrated the consensus shreds from all assemblers and a subset of the long-insert read pairs using parallel Phrap (11, 12). Possible misassemblies were corrected and some gap closures accomplished with PCR, manual editing in Consed, and in-house scripts (13).

Automatic annotation for each genome utilized an Ergatis-based workflow with minor manual curation. Each genome is available in the NCBI, and raw data can be provided upon request. In-depth comparative analyses of these and other genomes are under way and will be published in subsequent reports.

### Nucleotide sequence accession numbers.

The genome accession numbers to public databases are listed in Table 1.

**TABLE 1:** Listing of *Yersinia pestis* strains, their accession numbers, and genome sizes in this data set

Code	Strain	Country of origin	Accession no.^a^	Assembly status	Size (% G+C)^b^
BYA	1412	Georgia	CP006783 (Chr) CP006780 (pCD) CP006779 (pMT)	Finished	4,733,482 (47.6)
CAY	1413	Georgia	CP006762 (Chr) CP006761 (pCD) CP006760 (pMT)	Finished	4,736,923 (47.6)
DYA	1670	Georgia	AYLR00000000 (unscaffolded)	Improved high-quality draft	4.71 Mb (est) (47.6)
FKY	790	Kyrgyzstan	CP006806 (Chr) CP006807 (pMT) CP006808 (pPCP)	Finished	4,785,488 (47.6)
PYA	1522	Armenia	CP006758 (Chr) CP006757 (pCD) CP006756 (pMT)	Finished	4,738,644 (47.6)
RYP	2944	Russian Federation	CP006792 (Chr) CP006791 (pCD) CP006790 (pMT) CP006793 (pPCP)	Finished	4,672,740 (47.5)
VIP	3067	Georgia	CP006754 (Chr) CP006753 (pCD) CP006752 (pMT)	Finished	4,736,090 (47.5)
WYP	8787	Georgia	CP006748 (Chr) CP006747 (pCD) CP006746 (pMT)	Finished	4,675,987 (47.6)
YAE	3770	Georgia	CP006751 (Chr) CP006750 (pCD) CP006749 (pMT)	Finished	4,735,667 (47.6)
YPY	14735	Armenia	AYLS00000000 (unscaffolded)	Improved high-quality draft	4.69 Mb (est) (47.6)
ZPY	1045	Azerbaijan	CP006794 (Chr) CP006795 (pCD) CP006797 (pMT) CP006796 (pPCP)	Finished, noncontiguous (1 gap)	4.68 Mb (est) (47.5)

a Chr, chromosome; pCD, pMT, and pCP are plasmids.

^b^ Values for the genome sizes are shown in bp, unless otherwise indicated. est, estimated.
